# PD-1 blockade attenuates surgery-mediated immunosuppression and boosts Th1 immunity perioperatively in oesophagogastric junctional adenocarcinoma

**DOI:** 10.3389/fimmu.2023.1150754

**Published:** 2023-06-09

**Authors:** Maria Davern, Caoimhe Gaughan, Fiona O’ Connell, Brendan Moran, Eimear Mylod, Andrew D. Sheppard, Sinead Ramjit, Jasmine Yun-Tong Kung, James J. Phelan, Matthew G. Davey, Eanna J. Ryan, Christine Butler, Laura Quinn, Claudine Howard, Emily Tone, Eimear Phoenix, Waqas T. Butt, Niamh Lynam-Lennon, Stephen G. Maher, Narayanasamy Ravi, Claire L. Donohoe, John V. Reynolds, Joanne Lysaght, Noel E. Donlon

**Affiliations:** ^1^ Cancer Immunology and Immunotherapy Group, Department of Surgery, Trinity St. James’s Cancer Institute, Trinity Translational Medicine Institute, Dublin, Ireland; ^2^ Department of Medical Oncology, Dana-Farber Cancer Institute, Harvard Medical School, Boston, MA, United States; ^3^ Department of Surgery, Trinity St. James’s Cancer Institute, Trinity Translational Medicine Institute, St. James’s Hospital, Trinity College Dublin, Dublin, Ireland

**Keywords:** nivolumab and ipilimumab, PD-1, TIGIT, perioperative, Th1 immunity, immunosuppression, surgery, oesophageal adenocarcinoma

## Abstract

**Introduction:**

This timely study assesses the immunosuppressive effects of surgery on cytotoxic Th1-like immunity and investigates if immune checkpoint blockade (ICB) can boost Th1-like immunity in the perioperative window in upper gastrointestinal cancer (UGI) patients.

**Methods:**

PBMCs were isolated from 11 UGI patients undergoing tumour resection on post-operative days (POD) 0, 1, 7 and 42 and expanded *ex vivo* using anti-CD3/28 and IL-2 for 5 days in the absence/presence of nivolumab or ipilimumab. T cells were subsequently immunophenotyped *via* flow cytometry to determine the frequency of T helper (Th)1-like, Th1/17-like, Th17-like and regulatory T cell (Tregs) subsets and their immune checkpoint expression profile. Lymphocyte secretions were also assessed *via* multiplex ELISA (IFN-γ, granzyme B, IL-17 and IL-10). The 48h cytotoxic ability of vehicle-, nivolumab- and ipilimumab-expanded PBMCs isolated on POD 0, 1, 7 and 42 against radiosensitive and radioresistant oesophageal adenocarcinoma tumour cells (OE33 P and OE33 R) was also examined using a cell counting kit-8 (CCK-8) assay to determine if surgery affected the killing ability of lymphocytes and whether the use of ICB could enhance cytotoxicity.

**Results:**

Th1-like immunity was suppressed in expanded PBMCs in the immediate post-operative setting. The frequency of expanded circulating Th1-like cells was significantly decreased post-operatively accompanied by a decrease in IFN-γ production and a concomitant increase in the frequency of expanded regulatory T cells with an increase in circulating levels of IL-10. Interestingly, PD-L1 and CTLA-4 immune checkpoint proteins were also upregulated on expanded Th1-like cells post-operatively. Additionally, the cytotoxic ability of expanded lymphocytes against oesophageal adenocarcinoma tumour cells was abrogated post-surgery. Of note, the addition of nivolumab or ipilimumab attenuated the surgery-mediated suppression of lymphocyte cytotoxicity, demonstrated by a significant increase in tumour cell killing and an increase in the frequency of Th1-like cells and Th1 cytokine production.

**Conclusion:**

These findings support the hypothesis of a surgery-mediated suppression in Th1-like cytotoxic immunity and highlights a rationale for the use of ICB within the perioperative setting to abrogate tumour-promoting effects of surgery and ameliorate the risk of recurrence.

## Introduction

Oesophagogsatric junctional adenocarcinoma (OGJ) is an aggressive malignancy with a high propensity to metastasise (1). Clinical presentation of OGJ is often at the more advanced stages due to its indolent nature (1). The current treatment remains surgical en bloc resection, with multimodal perioperative therapy namely perioperative FLOT chemotherapy or preoperative CROSS chemoradiotherapy (2). FLOT treatment comprises 5-FU (anti-metabolite), oxaliplatin (platinum agent) and docetaxel (taxane) consisting of 4 neoadjuvant (pre-surgery) cycles and 4 adjuvant (post-surgery) cycles (2). The CROSS chemoradiotherapy regimen includes neoadjuvant carboplatin (platinum agent) and paclitaxel (taxane) with concomitant daily 1.8 Gy doses of radiotherapy with a cumulative dose of 41.4 Gy (2). The 5-year survival rate for OGJ cancer patients is between 20-30%, with approximately 70% of patients failing to achieve a complete pathologic response to neoadjuvant treatment (no viable tumour cells at time of surgical tumour resection) (3). And of the 30% that experience a complete pathologic response to treatment, up to 60% of those patients experience tumour recurrence within 5 years. In addition, of the patients who are prescribed FLOT perioperative treatment, only 33% of patients actually receive the complete 8 cycles of FLOT chemotherapy due to therapy-associated toxicities meaning that the majority of patients do not complete their anti-cancer treatment (4). The high tumour recurrence rate post-surgery in conjunction with the substantial dose limiting toxicities of current first-line adjuvant therapies highlights the clinical need to identify novel adjuvant therapies for OGJ cancer patients (4).

A recent study by Donlon et al, profiled systemic immunity in OGJ cancer patients perioperatively *via* flow cytometry immunophenotyping immune checkpoint expression profiles of circulating T cells and circulating soluble Th1, Th2, Treg, pro-inflammatory and pro-angiogenic factors in peripheral circulation (5). These findings highlighted the immunosuppressive effects of major oncologic surgery in OGJ cancer patients whereby surgery dampened systemic Th1 immune responses and promoted an immunosuppressive Th2 systemic immune response and increased circulating pro-angiogenic factors (5). These interesting findings suggest that the wound healing response induced by surgical tumour resection (Th2-like and regulator T cell-like) may be promoting metastatic dissemination and tumour recurrence and inhibiting cancer immune surveillance *via* dampening of the Th1-like immune response (5). However, it remains unknown if surgery dampens the cytotoxic immune response suppressing killing ability of lymphocytes. In this study we hypothesized that based on the findings from our previous study that surgery inhibits the cytotoxic ability of patient-derived lymphocytes and perhaps the use of immune checkpoint blockade (ICB) may abrogate these surgery-induced immunosuppressive effects. Recent clinical data from the Checkmate 577 trial, which led to the FDA approval of nivolumab for treating OGJ cancer patients in the adjuvant setting with residual disease, demonstrated that nivolumab significantly increased progression-free survival compared with the placebo arm (saline arm) (22.4 months versus 11 months) (6). Collectively, a rationale is highlighted to utilize immunotherapy in the perioperative surgical window to harness the immunomodulatory effects of surgery and promote anti-tumour Th1 responses. Therefore, our timely study aims to assess the therapeutic potential for incorporating ICB in the perioperative setting to boost Th1 cytotoxic immunity.

## Methods

### Patient cohort

12 patients undergoing surgical resection of OGJ tumours in St James’s Hospital Dublin and the Beacon Hospital, Dublin from April 2021 to July 2021 were recruited for this study. Inclusion criteria included: newly diagnosed patients with cancers of the upper gastrointestinal tract who may or may not have received neoadjuvant chemoradiotherapy prior to their surgery. Exclusion criteria included patients with metastasis. Based on power calculations we estimated a cohort size of 10 patients would be required to detect statistical significance therefore, we recruited a total of 12 patients. Whole blood was obtained from patients on the day of surgical tumour resection prior to surgery (before anaesthesia): post-operative day (POD) 0 (12 patients), 1 day post-surgery (POD 1) (12 patients), 7 days post-surgery (POD 7) (10 patients) and 6 weeks post-surgery (POD 42) (4 patients). Some patients were unavailable to follow up with for non-medical reasons. The cohort consisted of 7 males and 5 females, with an average age of 65.8 years. The patient demographics are detailed in **Table 1**.

**Table 1:** Patient demographics and clinical data.

Age (years)	65.8
**Sex ratio (M:F)**	7:5
Diagnosis (no. patients)	
**OSCC**	4
**Adenosquamous carcinoma**	1
**OGJ**	5
**Gastric adenocarcinoma**	2
Pathological tumour stage (no. patients)	
**T0**	0
**T1**	6
**T2**	0
**T3**	6
**T4**	0
Pathological nodal status (no. patients)	
**Positive**	5
**Negative**	7

### Ethical approval

Ethical approval was granted from the St. James’s Hospital Ethics Committee. All samples were collected with prior informed written consent for sample and data acquisition from patients attending St. James’s Hospital and the Beacon Hospital. This study was carried out in accordance with the World Medical Association’s Declaration of Helsinki guidelines on medical research involving human subjects. Patient samples were pseudonymised to protect the privacy rights of the patients.

### Serum and plasma isolation

Serum and plasma samples were obtained from patients on POD 0, 1, 7, 42, respectively. Whole blood samples were collected in BD Bioscience EDTA vacutainer blood tubes and were centrifuged at 3,000 RPM at room temperature. The plasma and serum were removed and stored at -80°C for future experimentation.

### Cell lines

Isogenic radiosensitive (OE33 P) and radioresistant (OE33 R) OGJ tumour cells were cultured in Roswell Park Memorial Institute 1640 Medium with 2 mM L-glutamine (ThermoFisher Scientific, Ireland) and supplemented with 1% (v/v) penicillin-streptomycin (50 U/ml penicillin 100μg/ml streptomycin) and 10% (v/v) foetal bovine serum (FBS) (ThermoFisher Scientific, Ireland) (complete RPMI). Cells were detached using trypsin-EDTA (Gibco, USA). Mycoplasma testing was routinely carried out by PCR every three months.

### Peripheral blood mononuclear cell isolation

Peripheral blood mononuclear cells (PBMCs) were isolated by density centrifugation using Ficoll-Paque (GE Healthcare, USA). Whole blood was diluted 1 in 2 with phosphate-buffered saline (PBS, Gibco, USA) and layered onto 15 ml of Ficoll-Paque™ (GE Healthcare, USA) and centrifuged at 2000 rpm for 25 minutes with the brake turned off. The buffy coat layer containing PBMCs was removed, washed with PBS (Gibco, USA) and resuspended at a density of 1x 10(6) cells/ml in complete RPMI with recombinant human IL-2 (10 units/ml) (ImmunoTools, Germany).

### Concomitant T cell activation and immune checkpoint blockade

A sterile 6-well plate was coated with 10 µg/ml of goat anti-mouse IgG (Sigma, USA) and 10 µg/ml of anti-CD3/anti-CD28 diluted in PBS (BioLegend, USA). PBMCs were seeded at a density of 1x 10(6) cells/ml in 2 ml of media with 10 IU of recombinant IL-2 cytokine (Biolegend, USA) in anti-CD3/28 pre-coated wells. Nivolumab and ipilimumab were each added to one well each at a concentration of 10 µg/ml. Nivolumab and Ipilimumab were kindly donated by the Beacon Hospital. Vehicle-treated PBMCs were treated with an isotype control antibody (IgG4,10 µg/ml, Biolegend, USA). Plates were incubated at 37°CC, 5% CO_2_ for 5 days. The media was topped up with an additional 1 ml of media every 2 days containing fresh IL-2 (10 IU) and 10 ug/ml of isotype control, nivolumab or ipilimumab. On day 5 of the T cell expansion a total of 6 ml of media was present in each well which contained the lymphocytes which were activated in the presence of a vehicle, nivolumab or ipilimumab. 1 ml of supernatant was harvested from each well and stored at -80°C for future experimentation on day 5 of the T cell expansion protocol.

### Co-culture with cancer cells

OE33 P cells and OE33 R cells were seeded at density of 1 x 10^4^ in 100 μl/in a flat 96 well plate in complete RPMI and were allowed adhere overnight at 37 °C, 5% CO_2_. The next day the media was replaced with 50 μl of complete RPMI and 50 µl of media only (untreated wells) or 50 µl of vehicle-, nivolumab- or ipilimumab-expanded lymphocytes were added at a density of 1 x 10^5^ cells in 50 µl of complete RPMI to achieve an effector to target ratio (E:T) of 10:1. Expanded lymphocytes were also cultured alone to account for the absorbance of lymphocytes using the CCK-8 assay. Cells were left to incubate for 48h at 37°CC, 5% CO_2_.

### Flow cytometry staining

Vehicle-, nivolumab- or ipilimumab-expanded lymphocytes were stained with zombie NIR viability dye, CD8-BV421, CD4-BV510, CD3-FITC, CXCR3-PE, CCR6-APC, CTLA-4-PerCPCy5.5, TIGIT-PE/Cy7, PD-L1-PerCPCy5.5, PD-1-PECy/7, CD25-FITC and FOXP3-BV421 (Biolegend, USA). Intracellular FoxP3 permeabilization and fixation buffer was used for FoxP3 staining (Biolegend, USA). Cells were acquired using the BD FACs Canto II (BD Biosciences) using Diva software and analysed using FlowJoTM v10.7.

### Cell counting kit-8 assay

Following a 48h co-culture of lymphocytes with OE33 P and OE33 R tumour cells, the viability of tumour cells was assessed using a cell counting kit (CCK-8, Sigma, USA) viability assay. 5 µl of CCK-8 solution was added to each well for 1h at 37°CC, 5% CO_2_. The optical density was measured using the Versa Max microplate reader (Molecular Devices, Sunnyvale, CA, USA) at 450 nm and 650 nm (reference wavelength). Formula: (viability OE33 cell-lymphocyte co-culture)-(viability PBMCs alone)/(viability untreated OE33 cells alone) × 100 = % live cells.

### Quantification of serum and lymphocyte supernatant immune proteins

Serum samples were processed according to MSD (Meso Scale Discovery) multiplex protocol. To assess interferon-gamma (IFN-γ), granzyme B (GrB), interleukin-10 (IL-10) and IL-17 cytokines, a custom 4 spot U-PLEX ELISA kit was used (Meso Scale Diagnostics, USA). Serum samples were diluted 1:2 for analysis and lymphocyte supernatants were run undiluted. All assays were run as per manufacturer’s recommendation, an overnight supernatant incubation protocol was used for all assays. Analyte concentrations were calculated using Discovery Workbench software (version 4.0). Values outside the kits limit of detection were not reported. For the purpose of this study these immune proteins were compared between patients who had lower than median IFN-γ levels versus higher than median IFN-γ levels to determine if there were particular proteins that were differentially expressed based on circulating levels of IFN-γ. We chose IFN-γ in this instance, as patients with higher circulating levels of IFN-γ in their serum on POD 0 also had a greater nivolumab-mediated increase in IFN-γ by expanded lymphocytes *ex vivo*. Based on this observation we sought to investigate what might be different in the circulating factors between patients with high versus low IFN-γ in their serum.

### Statistical analysis

Statistical analysis was performed using GraphPad Prism 9.1.1 software (GraphPad Prism, San Diego, California). Significance level of p ≤ 0.05 was used for all analyses. CCK-8 data and flow cytometry data were analysed using a two-way ANOVA statistical analysis. Spearman correlative analysis was used to assess correlations.

## Results

### Th1 immunity is dampened in expanded PBMCs in the immediate post-operative setting

**Figure 1:**
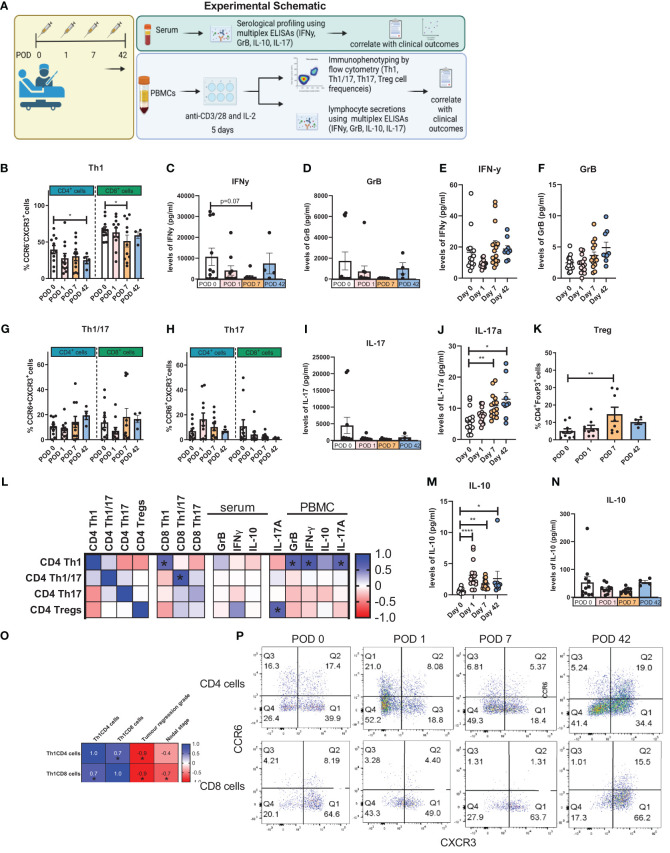
The Th1 immune response was dampened in expanded PBMCs in the immediate postoperative setting. PBMCs were isolated from peripheral blood of cancer patients on POD 0 (n=12), POD 1 (n=12), POD 7 (n=11) and POD 42 (n=4) and activated *ex vivo* for 5 days using plate bound anti-CD3/28 and recombinant IL-2 **(A)**. The frequency of expanded Th1 (CXCR3^+^CCR6^-^) **(B)**, Th1/17 (CXCR3^-^CCR6^-^) **(G)** and Th17 (CXCR3^+^CCR6^+^) **(H)** CD3^+^CD4^+^ and CD3^+^CD8^+^ cells and Treg cells (CD4^+^FOXP3^+^) **(K)** was assessed by flow cytometry. The soluble levels of IFN-γ **(C, E)**, GrB **(D, F)**, IL-17 **(I, J)** and IL-10 **(M, N)** were measured in the serum from whole blood and in the supernatant from expanded PBMCs on POD 0, 1, 7 and 42. **(L)** shows the correlation values between cytokine production and the frequency of expanded lymphocytes following a 5-day expansion. **(O)** shows the significant correlation values between T cell subset frequencies post-5-day expansion with clinical characteristics. **(P)** shows representative dot plots for the frequency of Th1, Th17 and Th1/17 cells. Data presented as mean ± SEM. Two-way ANOVA for **(B–K)** and **(M, N)**. Spearman correlation used for non-parametric correlative analysis for **(L, O)**. The r^2^ value indicates the correlation value. A red square indicates a positive correlation i.e. R^2^>0. A blue square indicates a negative correlation i.e. R^2^<0. An asterisk depicts the significant correlations i.e. r^2^ value has a p=0.05.

The balance between Th1 and regulatory T cell (Treg) immune responses has a profound impact on anti-cancer immunity. Additionally, Th17 immune responses can have either a tumour-promoting or tumour-suppressive effect, which is often context dependent. Therefore, this study assessed the frequency of expanded circulating Th1-, Th17- and Th1/17-like CD3^+^CD4^+^ and CD3^+^CD8^+^ cells from OGJ cancer patients who underwent surgical tumour resection to determine how major oncologic surgery affects systemic Th1, Treg and Th17 immunity (**Figure 1A**).

Overall, systemic Th1 immune responses appeared to be dampened in the immediate post-operative setting in OGJ cancer patients paralleled by an increase in regulatory T cell immunity. The frequency of expanded circulating CD4^+^CCR6^-^CXCR3^+^ Th1-like cells was significantly decreased on POD 42 compared with POD 0 (**Figures 1B, P**, 25.1 ± 4.8 vs. 39.5 ± 5.6%, p=0.05). Similarly, the frequency of expanded circulating CD8^+^CCR6^-^CXCR3^+^ Th1-like cells was significantly decreased on POD 7 compared to POD 0 (**Figures 1B, P**, 51.7 ± 8.1 vs. 66.7 ± 3.9%, p=0.02). This was accompanied by a decrease in IFN-γ production from expanded PBMCs on POD 7 compared with POD 0, that trended toward statistical significance, indicative of a Th1 suppressed immune response in the post-operative setting (**Figure 1C**, 1,081 ± 661.6 vs. 10,682 ± 4,156%, p=0.07). We did not observe any significant changes in the production of GrB in the secretome of expanded lymphocytes post-operatively compared with pre-operative levels (**Figure 1D**). The levels of IFN-γ and GrB in the serum of whole blood was not significantly altered post-operatively compared with pre-operative levels (**Figures 1E, F**). Although we observed a significant increase in the circulating levels of IL-17 in the serum of whole blood post-operatively on POD 7 and POD 42 compared with pre-operative levels (**Figure 1J**), the frequency of expanded Th1/17 and Th17 cells remained unchanged as did the levels of IL-17 secretions from expanded lymphocytes (**Figures 1G–I**). Interestingly, we identified a significant increase in the levels of circulating IL-10 in the serum of whole blood in OGJ cancer patients post-operatively on POD 1, 7 and 42 highlighting that the immunosuppressive arm of the immune system was engaged post-oesophagectomy, which may be detrimental in the elaboration of anti-cancer Th1 immune responses and cancer immune surveillance, conversely promoting tumour recurrence and dissemination in the post-operative adjuvant setting (**Figure 1M**, 3.1 ± 0.5, 1.7 ± 0.1, 2.6 ± 1.2 vs. 0.1 ± 0.6%, p<0.001, p,0.001 and p=0.009). Induction of immunosuppression post-surgery was further underlined by a significant increase in the frequency of expanded regulatory T cells (Tregs) by POD 7 (**Figure 1K**, 14.7 ± 4.0 vs. 5.1 ± 1.2%, p=0.04). We did not detect any differences in the levels of IL-10 in the supernatant of expanded PBMCs (**Figure 1N**). Furthermore, we observed that the frequency of expanded CD4^+^ and CD8^+^ Th1-like cells following 5-day T cell activation was significantly correlated with a worse pathologic response to neoadjuvant treatment (r= -0.9, p=0.03). The presence of nodal metastasis also significantly correlated with the frequency of expanded CD8^+^ Th1-like cells following 5-day T cell activation (r= -0.6, p=0.03, respectively). This might indicate that patients who responded poorly to treatment and had more advanced disease may have a dampened Th1 immune response as the expansion of Th1-like cells *ex vivo* was attenuated. The frequency of expanded T helper subsets was also correlated with the levels of T helper-associated cytokines in the supernatant from expanded PBMC cultures and in peripheral circulation of cancer patients (**Figure 1L**). Not surprisingly, the levels of Th1-associated cytokines such as GrB and IFN-γ correlated with the frequency of expanded Th1-like CD4 cells *ex vivo* (**Figure 1L**). In addition, the levels of pro-inflammatory cytokine 1L-17 in the supernatant of expanded PBMCs also positively correlated with the levels of expanded Th1-like CD4 cells *ex vivo* (**Figure 1L**).

It was observed that the frequency of expanded Th1-like CD8^+^ cells negatively correlated with nodal stage i.e. patients who had higher levels of CD8^+^ Th1-like cells had a decreased nodal stage or fewer number of lymph nodes with metastasis. This suggests that in patients with more advanced stage disease CD8 anti-tumour immunity may be blunted (**Figure 1O**). Similarly, patients who had higher levels of expanded CD4^+^ or CD8^+^ Th1-like immune cells had an improved pathologic response to treatment with lower tumour regression scores (**Figure 1O**). Again underpinning the importance of Th1 immune responses in disease progression and treatment response.Several studies have highlighted major differences between female versus male immunity and how important it is on the efficacy of cancer treatments and responses. Therefore, we carried out statistical analysis between the five female patients and seven male patients to determine if there were any statistically significant differences in any of the immunological parameters between the two groups which included the frequency of circulating immune cells, levels of 5-day expanded lymphocyte-secreted cytokines and immune checkpoint expression of profiles of the different T cell subsets (**Figure S1**). The two groups were comparable however, recognizing the heterogeneity of patient samples and the low number of numbers in both groups, there may be a statistically significant difference if more patient samples were included.

In summary, our findings suggest that an IL-10-driven immunosuppressive window is propagated by resection of OGJ tumours in the post-operative setting and this is accompanied by a dampening of a Th1 immune response, which is maintained *ex vivo* in expanded PBMCs. Overall, this data highlights the need to introduce immunotherapies in the post-operative setting to exploit this surge in immune activation and skew it towards an anti-cancer Th1 immune response to enhance cancer immune surveillance and reduce the high tumour recurrence rate in the adjuvant setting.

### PD-L1 expression was significantly increased on the surface of expanded Th1-like cells in the immediate postoperative setting

**Figure 2:**
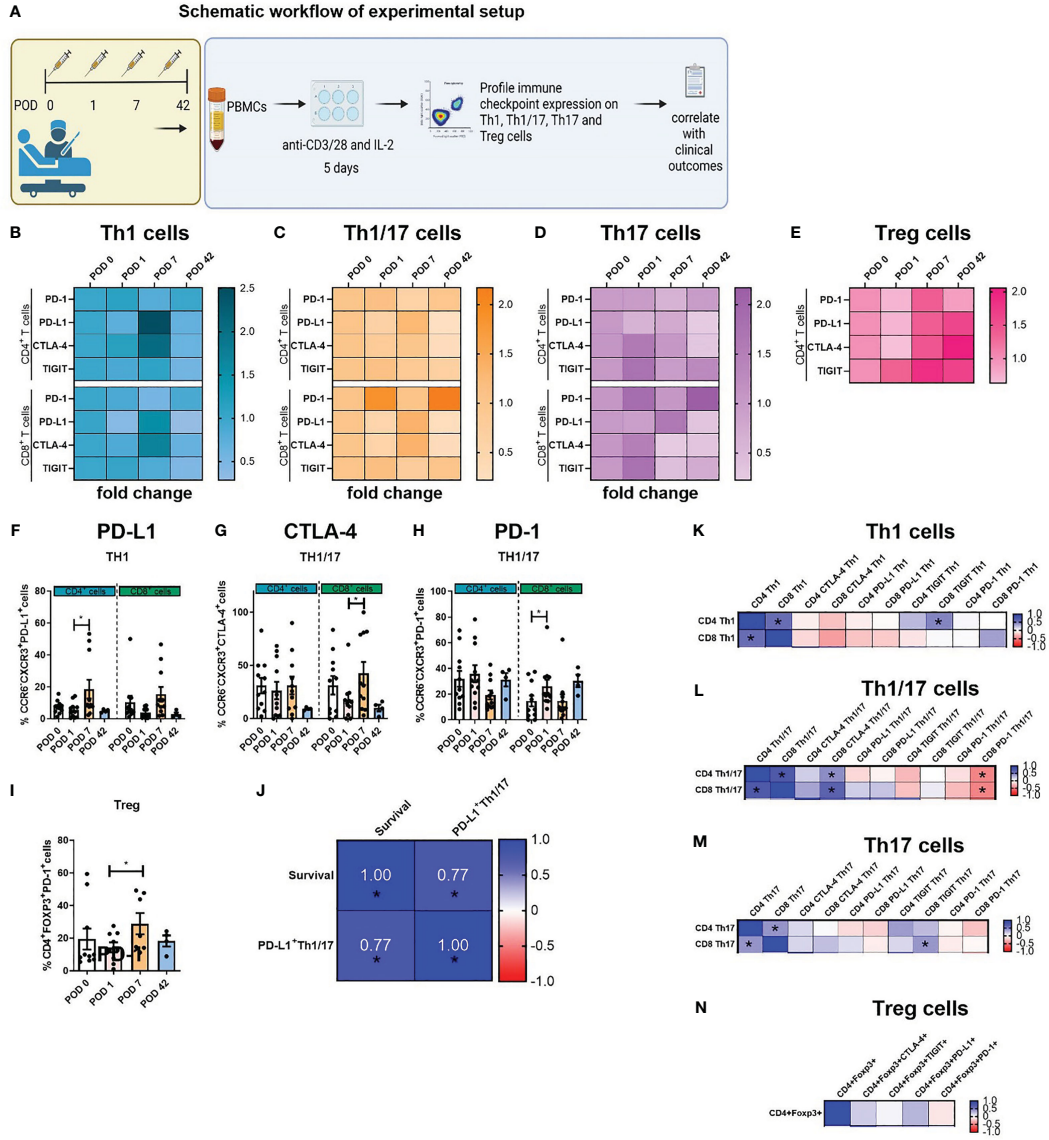
Expression of PD-L1 on the surface of expanded Th1-like cells was significantly increased post-operatively. PBMCs were isolated from peripheral blood of Cancer patients on POD 0 (n=11), POD 1 (n=11), POD 7 (n=11) and POD 42 (n=4) and activated *ex vivo* for 5 days using plate bound anti-CD3/28 and recombinant IL-2 **(A)**. The immune checkpoint expression profile of expanded Th1 (CXCR3^+^CCR6^-^) **(B)**, Th1/17 (CXCR3^-^CCR6^-^) **(C)** and Th17 (CXCR3^+^CCR6^+^) **(D)** CD4^+^ and CD8^+^ cells and Treg cells (CD4^+^FOXP3^+^) **(E)** is displayed as a fold change in expression that is normalised to the levels of immune checkpoints on POD 0. The percentage of specific T cell subsets within the entire T cell population are shown and was determined by flow cytometry. **(F)** Shows the percentage of Th1-like cells expressing PD-L1and, **(G, H)** highlight percentage of expanded Th1/17-like cells expressing CTLA-4 and PD-1, respectively. **(I)** Highlights the percentage of expanded Treg cells expressing PD-1. **(K–N)** Presents the correlation values between the frequency of expanded circulating CD4^+^ and CD8^+^ T helper subsets and their respective IC expression profiles for Th1-like cells, Th1/17-like cells, Th17-like cells and Treg cells. **(J)** Showcases the significant correlations between the frequency of IC expression on T helper subsets with clinical parameters in cancer patients. Data presented as mean ± SEM. Two-way ANOVA analysis for F-I. Spearman correlation used for non-parametric correlative analysis for **(J–N)**. The r^2^ value indicates the correlation value. A red square indicates a positive correlation i.e. R^2^>0. A blue square indicates a negative correlation i.e. R^2^<0. An asterisk depicts the significant correlations i.e. r^2^ value has a p=0.05.

Due to the reduction in expanded circulating Th1-like cells in the immediate post-operative period, we investigated whether there was a corresponding increase in the expression of inhibitory immune checkpoint proteins on the surface of Th1-like cells (**Figure 2B**), Th1/17-like (**Figure 2C**), Th17-like cells (**Figure 2D**) and Treg cells (**Figure 2E**) as outlined in the experimental schematic in **Figure 2A**. Expression of TIGIT on expanded circulating T cells did not significantly change (**Figure 2**). Interestingly, the frequency of PD-L1 expression on expanded circulating CD4^+^CCR6^-^CXCR3^+^ Th1-like cells was significantly increased on POD 7 compared to POD 1 (**Figure 2F**, 18.55 ± 5.8 vs. 7.4 ± 1.2%, p=0.04). The frequency of CTLA-4 expression on expanded circulating CD8^+^CCR6^-^CXCR3^+^ Th1/17-like cells was significantly increased on POD 7 compared to POD 1 (**Figure 2G**, 42.4 ± 10.7 vs. 17.8 ± 5.7%, p=0.04). The frequency of PD-1 expression on expanded circulating Treg cells was significantly increased on POD 7 compared to POD 1 (**Figure 2I**, 28.8 ± 6.5 vs. 19.4 ± 6.5%, p=0.04). Finally, the frequency of PD-1 expression on expanded circulating CD8^+^CCR6^-^CXCR3^+^ Th1/17-like cells was significantly increased on POD 7 compared to POD 1 (**Figure 2H**, 26.2 ± 5.2 vs. 14.7 ± 4.2%, p=0.04). Moreover, the frequency of PD-L1^+^ Th1/17-like cells following a 5-day activation correlated with worse overall survival (**Figure 2J**). The correlation between the frequencies of expanded Th subsets and the expression of immune checkpoints on surface of Th1, Th1/17, Th17 and Treg cells was also carried out and depicted in **Figures 2K–N**.

### Single agent nivolumab abrogated the surgery-induced dampening of Th1 anti-tumour immune responses in a subset of patients

**Figure 3:**
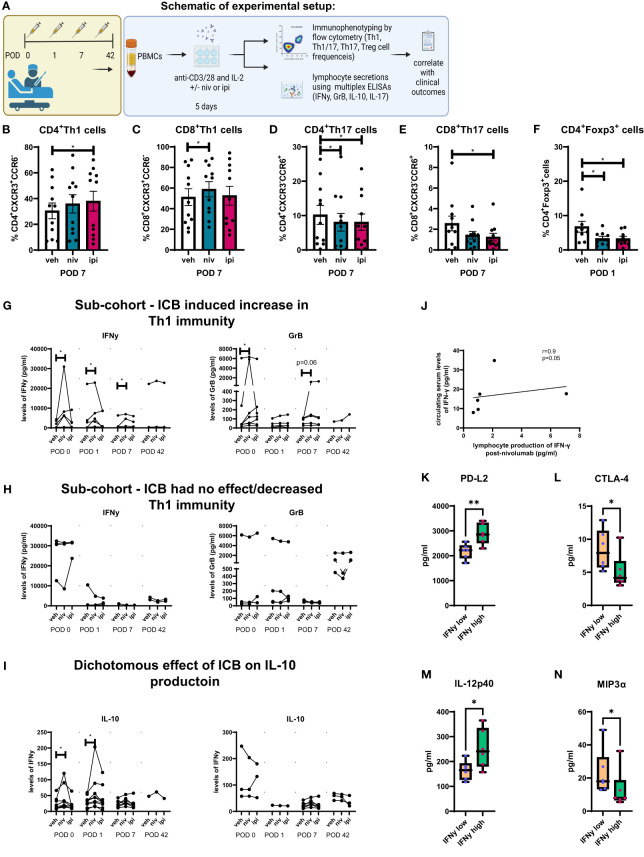
Single agent nivolumab abrogated the surgery-induced dampening of anti-tumour Th1 immune responses in a subset of patients. **(A)** Experimental schematic. PBMCs were isolated from peripheral blood of Cancer patients on POD 0 (n=11), POD 1 (n=11), POD 7 (n=11) and POD 42 (n=4) and activated *ex vivo* for 5 days using plate bound anti-CD3/28 and recombinant IL-2 in the presence or absence of nivolumab (niv) (10 µg/ml) or ipilimumab (ipi) (10 µg/ml). The frequency of expanded Th1 (CXCR3^+^CCR6^-^), Th17 (CXCR3^-^CCR6^-^) and Th1/17 (CXCR3^+^CCR6^+^) CD4^+^ and CD8^+^ cells and Treg cells (CD4^+^FOXP3^+^) was assessed by flow cytometry. The levels of IFN-γ, GrB and IL-10 were measured in the supernatant from expanded PBMCs on POD 0, 1, 7 and 42. **(B–F)** Displays the effect of nivolumab and ipilimumab treatment on the frequency of expanded CD4^+^ and CD8^+^ CXCR3^+^CCR6^-^ Th1-like cells on POD 7, the frequency of expanded CD4^+^ and CD8^+^ CXCR3^+^CCR6^+^ Th17-like cells on POD 7 and the frequency of Treg cells on POD 1. **(G)** Presents the sub-cohort of patients (7/10) whose PBMCs increased their IFN-γ and GrB production following nivolumab treatment. **(H)** Presents the sub-cohort of patients whose PBMCs had no increase or showed a decrease in their IFN-γ and GrB production following nivolumab treatment. **(I)** Shows two sub-cohorts of patients whose PBMCs had an increase (7/10) or a decrease (3/10) in their IL-10 production following nivolumab treatment. **(J)** Correlation plot showing a significant correlation between the effect of nivolumab on lymphocyte secretion of IFN-γ following a 5-day T cell activation with the circulating levels of IFN-γ in the serum of matched patients on POD 0. **(K–N)** Shows the soluble circulating factors that were significantly altered in the serum of patients who had higher than median levels of circulating IFN-γ compared with patients who had lower than median levels. All data presented as mean ± SEM. Two-way ANOVA. Correlative analysis: Spearman. The r^2^ value indicates the correlation value. A red square indicates a positive correlation i.e. R^2^>0. A blue square indicates a negative correlation i.e. R^2^<0. An asterisk depicts the significant correlations i.e. r^2^ value has a p=0.05.

Our findings have identified an immunosuppressive window created by cancer surgery in the post-operative setting, which may likely contribute to the suppression of cancer immune surveillance and tumour dissemination and recurrence. Therefore, these results highlight the urgent clinical need to identify therapeutics that can counteract the propagation of immunosuppression mediated by surgery in the adjuvant setting. Subsequently, we explored the use of ICB namely, single agent anti-PD-1 and anti-CTLA-4 therapy to determine if these agents might enhance anti-tumour Th1 immunity post-operatively using our *ex vivo* PBMC expansion protocol (**Figure 3A**).

Ipilimumab significantly increased the frequency of expanded CD4^+^CCR6^-^CXCR3^+^ Th1-like cells compared with untreated cells on POD 7 (**Figure 3B**, 37.9 ± 7.6 vs. 30.4 ± 6.1%, p=0.03). In addition, nivolumab significantly increased the frequency of expanded CD8^+^CCR6^-^CXCR3^+^ Th1-like cells compared to untreated cells on POD 7 (**Figure 3C**, 58.8 ± 7.3 vs. 51.2 ± 8.1%, p=0.04). Both single agent nivolumab and ipilimumab decreased the frequency of expanded Th17-like cells on POD 7 (**Figures 3D, E**). Furthermore, nivolumab and ipilimumab significantly reduced the frequency of expanded Treg cells on POD 1 (**Figure 3F**, 3.3 ± 0.6, 3.2 ± 0.7% vs. 6.7 ± 1.5, p=0.04 and p=0.04). Additionally, we observed in a sub-cohort of 7/10 patients that single agent nivolumab significantly increased the production of IFN-γ by expanded PBMCs on POD 0, 1 and 7 (**Figure 3G**). Complementary findings demonstrated that GrB production was significantly enhanced by single agent nivolumab on POD 0 in the same sub-cohort of 7/10 patients (**Figure 3G**). Furthermore, expanded PBMCs from 3 patients did not exhibit an increase in IFN-γ or GrB levels (**Figure 3H**). Interestingly, in the sub-cohort of patients that experienced an enhancement in Th1 immunity in their nivolumab-expanded PBMCs a concomitant increase in IL-10 production was also noted on POD 0 and 1 in parallel, which may represent an immunosuppressive mechanism of resistance to nivolumab treatment (**Figure 3I**).

In our study we also conducted correlative analysis between the magnitude of response to ICB (magnitude of response characterised by alteration in PBMC secretion of IFN-γ in PBMC culture following 5 days of T cell activation) in expanded PBMCs with circulating serum proteins in cancer patients on POD 0, 1 and 7 to determine if there was a circulating factor that might indicate if the use of ICB could enhance Th1 immune responses in a patients PBMCs.

Given that ICB increased IFN-γ, GrB and IL-10 in only a sub-cohort of patients we wondered if there might be circulating factors in patients’ serum that might correlate with this favourable response to ICB (favourable response to ICB: nivolumab/ipilimumab-mediated increase in GrB or IFN-γ). Identifying any correlations between a favourable response to ICB *ex vivo* and circulating factors in patients’ serum might reveal a potential explanation behind why some patients’ PBMCs responded favourably to ICB whilst others did not. We performed this analysis by correlating the fold-change in nivolumab- and ipilimumab-induced alterations in IFN-γ, GrB and IL-10 produced by PBMCs with the levels of circulating IFN-γ, GrB and IL-10 in the serum of whole blood in matched patients on POD 0, 1 and 7 (**Figure 3J**). On POD 0 there was a positive correlation between the nivolumab-induced increase in IFN-γ production by PBMCs *ex vivo* with the circulating levels of IFN-γ in the serum of whole blood in cancer patients on POD 0. This correlation suggests that patients who have higher levels of circulating IFN-γ in their peripheral blood may possess a particular immune state that makes their PBMCs more amenable to nivolumab-mediated enhancement in Th1 immunity.

As the levels of IFN-γ in patients on POD 0 was the only mediator that significantly correlated with a favourable response to ICB, we next set out to understand what is different between the soluble immune factors in systemic circulation between patients who have higher circulating levels of IFN-γ versus patients with lower levels. This might contribute to an explanation as to why patients with higher IFN-γ levels possess PBMCs that are predisposed to experiencing a nivolumab-induced increase in Th1 immunity versus those that don’t respond in this way. We examined the difference in the levels of 35 soluble immune proteins in 10 patients with high versus low IFN-γ levels using the median level as a cut-off for high versus low. Of note only 4 soluble immune proteins were differentially expressed between patients with high versus low IFN-γ levels. In the sub-cohort of patients with higher levels of IFN-γ, there was a significant difference in the levels of circulating soluble immune checkpoint proteins, specifically these patients had significantly higher levels of soluble PD-L2 (**Figure 3K**, 2882 ± 170.6 vs. 2176 ± 121.4 pg/ml, p=0.008) and significantly lower levels of soluble CTLA-4 (**Figure 3L**, 5.11 ± 1.1 vs. 8.4 ± 1.2 pg/ml, p=0.04), which might explain why these patients responded better to nivolumab over ipilimumab. Moreover, in the patients with higher circulating levels of IFN-γ there was a significantly higher level of soluble IL-12p40, an important Th1-like cytokine indicating that these patients may have an already primed and ongoing anti-tumour immune response (**Figure 3M**, 252.6 ± 32.2 vs. 163.9 ± 15.43 pg/ml, p=0.04). Lastly, patients with higher circulating levels of IFN-γ also had a significantly lower circulating level of MIP-3α (**Figure 3N**, 12.9 ± 4.8 vs. 23.1 ± 5.6 pg/ml, p=0.04).

In summary, only a sub-cohort of patients possessed PBMCs that experienced a nivolumab-mediated increase in Th1 immunity measured by an increase in IFN-γ secretion by expanded PBMCs. These patients were enriched with higher levels of circulating IFN-γ, which might indicate that these patients had an ongoing anti-tumour immune response that was amenable to nivolumab-mediated enhancement in Th1 immunity.

**Figure 4:**
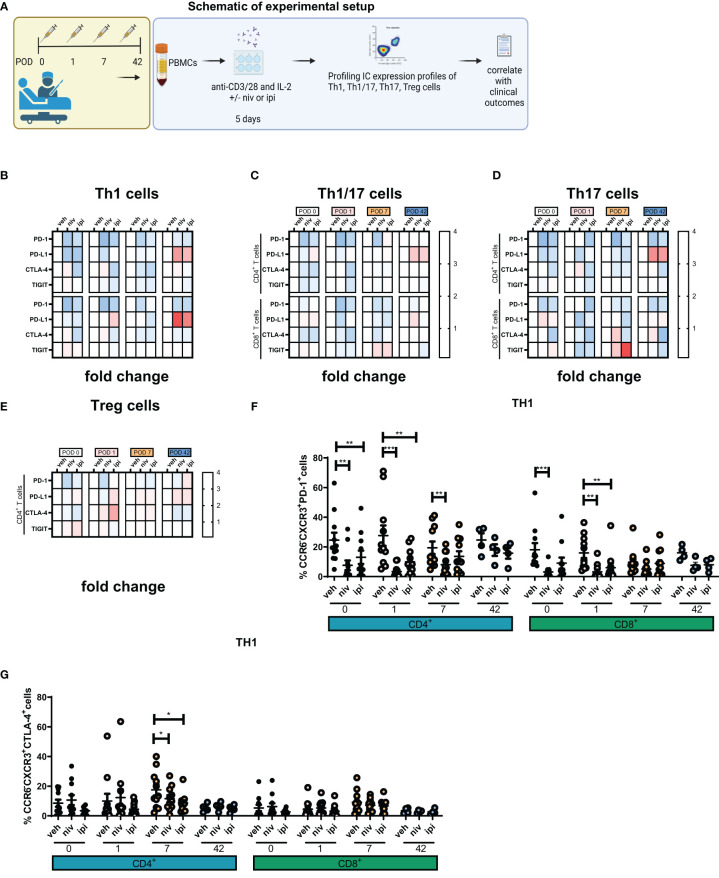
Single agent nivolumab and ipilimumab treatment significantly decreased the expression of PD-1 and CTLA-4 on the surface of expanded Th1-like cells *ex vivo*. PBMCs were isolated from peripheral blood of Cancer patients on POD 0 (n=11), POD 1 (n=11), POD 7 (n=11) and POD 42 (n=4) and activated *ex vivo* for 5 days using plate bound anti-CD3/28 and recombinant IL-2 in the presence or absence of nivolumab (niv) (10 µg/ml) or ipilimumab (ipi) (10 µg/ml) **(A)**. The IC expression profile of expanded Th1 (CXCR3^+^CCR6^-^) **(B)**, Th1/17 (CXCR3^+^CCR6^+^) **(C)**, Th17 (CXCR3^-^CCR6^-^) **(D)** and CD4^+^ and CD8^+^ cells and Treg cells (CD4^+^FOXP3^+^) **(E)** was assessed by flow cytometry. The percentage of T cell subsets positive for an immune checkpoint is summarized in a heat map format. **(F, G)** Display the effects of ICB on the expression of PD-1 and CTLA-4 on the surface of expanded PBMCs *ex vivo*. **(B–E)** In each heat map a value greater than 1 demonstrates ICB increased the percentage of cells positive for an immune checkpoint compared with the effect of the vehicle on that T cell subset in a particular POD timepoint sample be it POD 0, 1, 7 or 42. Similarly, a value less than 1 indicates a decrease and a value equal to 1 indicates no effect was observed. All data presented as mean ± SEM. Two-way ANOVA analysis for **(F, G)**. *p<0.05, **p<0.01, ***p<0.001.

In consideration of the observed increase in IC expression and subsequent reduction in Th1-like cells following surgery, we postulated that the use of ICB might attenuate the global increased expression of IC proteins contributing to the enhancement in Th1 immune responses (**Figure 4A**). Overall, nivolumab treatment significantly decreased PD-1 expression on the surface of T helper subsets as expected (**Figures 4B–G**). Surprisingly, nivolumab also decreased PD-L1 expression on the surface of Th1-like, Th1/17-like and Th17 like cells (**Figures 4B–E**). Moreover, CTLA-4 blockade significantly decreased CTLA-4 on the surface of T helper subsets but also surprisingly decreased the expression of PD-1 on the surface of Th-1-like, Th1/17-like and Th17-like cells (**Figures 4B–G**). Overall, treatment with ICB reduced the expression of IC proteins on Th1-like cells, which may be a contributing factor in the unleashing of anti-tumour Th1 immunity following ICB.

### The cytotoxic ability of expanded lymphocytes is impaired in the immediate postoperative setting, an effect which is attenuated by ICB

**Figure 5:**
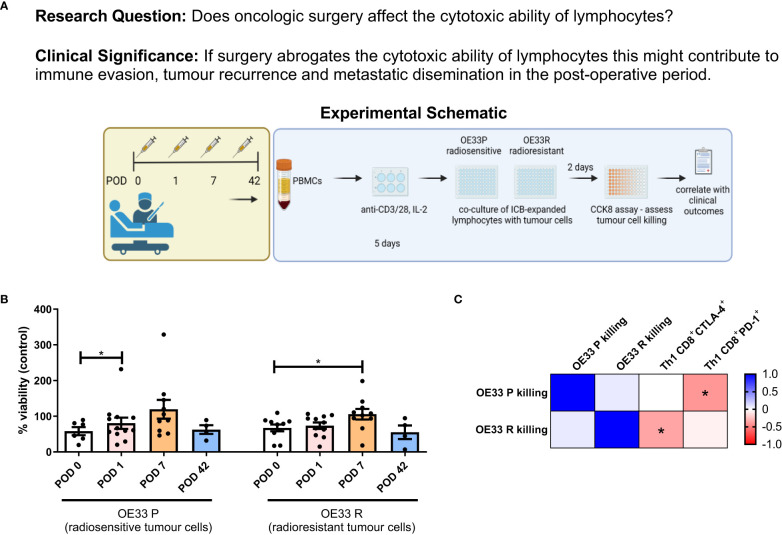
Lymphocyte-mediated killing of OE33 P and OE33 R cells is impaired in the immediate post-operative setting. **(A)** Experimental schematic. **(B)** OE33 P and OE33 R cells were co-cultured with PBMCs isolated from Cancer patients undergoing surgical tumour resection on POD 0 (n=7), 1 (n=10), 7 (n=10) and week 6 (n = 4) in an effector:target ratio (E:T) of 10:1 1 x 10^5^:1 x 10^4^for 48h. PBMCs were pre-activated using plate bound anti-CD3/28 and IL-2 for 5 days. A CCK8 assay was used to determine the viability of OE33 P and OE33 R cells. **(C)** Presents a correlation matrix between lymphocyte-mediated killing of OE33 P and OE33 R cells and the expression of PD-1 and CTLA-4 on the surface of expanded circulating CD8^+^ T cells that were present in the tumour cell:PBMC co-culture. A value greater than 0 (red) is a positive correlation and a value below zero (blue) is a negative correlation. Spearman correlation used for non-parametric correlative analysis. The r^2^ value indicates the correlation value. A red square indicates a positive correlation i.e. R^2^>0. A blue square indicates a negative correlation i.e. R^2^<0. An asterisk depicts the significant correlations i.e. r^2^ value has a p=0.05. Data were analysed using a two-way ANOVA and presented as percentages ± SEM.

Given the immunosuppressive milieu in the immediate postoperative setting, we investigated whether this would reduce the cytotoxic ability of expanded circulating lymphocytes to kill radiosensitive and radioresistant OGJ tumour cells (OE33 P and OE33 R isogenic cell line) (**Figure 5A**). Lymphocyte-mediated cytotoxicity of OE33 P cells was significantly reduced on POD 1 compared to POD 0 (**Figure 5B**, 80.44 ± 15.8 vs. 58.25 ± 11.4% p=0.04). Similarly, lymphocyte-mediated cytotoxicity of OE33 R cells was significantly reduced on POD 7 compared to POD 0 (**Figure 5B**, 105.7 ± 14.8 vs. 67.27 ± 9.5%, p=0.03). Overall, cancer surgery significantly impaired the cytotoxic ability of expanded circulating lymphocytes by POD 7.

**Figure 6:**
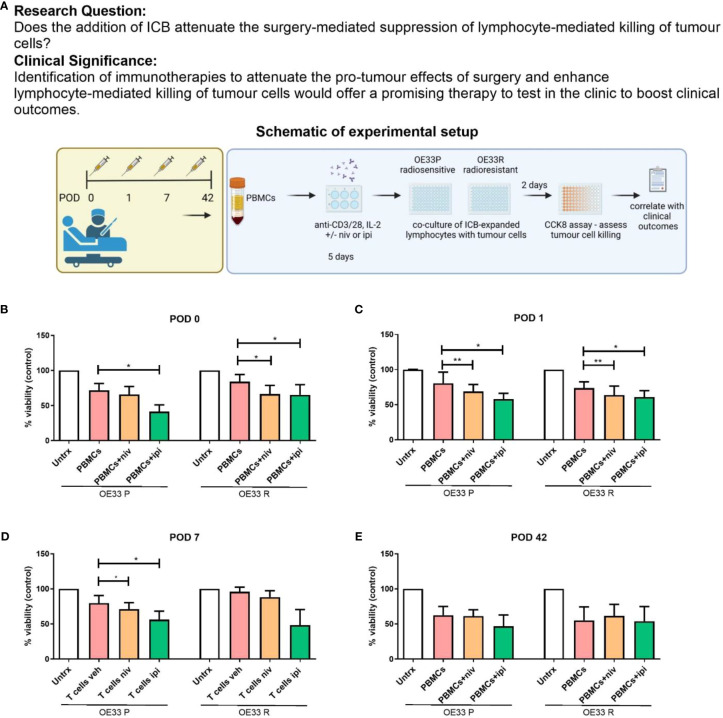
Nivolumab and ipilimumab enhanced lymphocyte-mediated killing of OE33 P and OE33 R cells in the immediate post-operative setting. **(A)** Experimental schematic. OE33 P and OE33 R cells were co-cultured with PBMCs isolated from cancer patients on POD 0 (n = 11) **(B)**, 1 (n = 11) **(C)**, 7 (n = 11) **(D)** and week 6 (n = 4) **(E)** in an E:T ratio of 10:1 (100,000:10,000) for 48h. PBMCs were pre-activated using plate bound anti-CD3/28 in the presence or absence of nivolumab (niv) (10 µg/ml) or ipilimumab (ipi) (10 µg/ml) for 5 days. A CCK8 assay was used to determine the viability of OE33P and OE33R cells. Two-way ANOVA statistical analysis for **(B–E)**. *p<0.05, **p<0.01.

Interestingly, lymphocyte-mediated killing of OE33 P cells was negatively correlated with the frequency of PD-1^+^CD8^+^ T cells suggesting that the PD-1 axis may be hindering the ability of expanded lymphocytes to kill tumour cells (**Figure 5C**). Additionally, lymphocyte-mediated killing of OE33 R cells was negatively correlated with the frequency of CTLA-4^+^CD8^+^ T cells suggesting that the CTLA-4 axis may be hindering the ability of expanded lymphocytes to kill tumour cells as well (**Figure 5C**). This prompted us to next investigate if the addition of anti-PD-1 ICB or anti-CTLA-4 ICB might attenuate the surgery-mediated suppression in lymphocyte killing of OE33 P and OE33 R tumour cells (**Figure 6A**).

Nivolumab significantly enhanced the killing of OE33 R cells compared with untreated cells on POD 0 (**Figure 6B**, 57.9 ± 15.9 vs. 71.43 ± 15.7%, p=0.04) and POD 1 (**Figure 6C**, 63.5 12.9 vs. 73.52 8.9%, p=0.03. Nivolumab also significantly enhanced the killing of OE33 P cells on POD 1 (**Figure 6C**, 63.5 ± 12.9 vs. 73.5 ± 8.9%, p=0.04) and POD 7 (**Figure 6D**, 71.1 ± 9.1 vs. 79.6 ± 10.6%, p=0.04). Ipilimumab significantly enhanced the killing of OE33 P cells compared with untreated cells on POD 0 (**Figure 6B**, 41.4 ± 9.6 vs. 71.6 ± 9.7%, p=0.02), POD 1 (**Figure 6C**, 60.7 ± 9.2 vs. 73.5 ± 8.9%, p=0.04) and POD 7 (**Figure 6D**, 56.1 ± 12.1 vs. 79.7 ± 10.6%, p=0.04). Likewise, Ipilimumab significantly enhanced the killing of OE33 R cells compared with untreated cells on POD 0 (**Figure 6B**, 51.4 ± 14.3 vs. 71.43 ± 15.7%, p=0.03) and POD 1 (**Figure 6C**, 60.7 ± 9.2 vs. 73.5 ± 8.9%, p=0.04). ICB did not significantly improve the ability of lymphocytes isolated from patients 6 weeks post-surgery in killing OE33 P or OE33 R tumour cells (**Figure 6E**). Overall, the use of ICB abrogated the surgery-induced reduction in the cytotoxic ability of expanded PBMCs against radiosensitive and radioresistant OGJ tumour cells *ex vivo*.

## Discussion

Surgery creates a unique immune environment with immunosuppressive, pro-inflammatory, pro-angiogenic and pro-proliferative effects (7–9). Combining immunotherapy with surgery to turn an immunosuppressive effect into a therapeutic opportunity is an attractive and rational strategy. In the case of cancer resection, this creates an ideal environment for the promotion of micrometastatic residual disease, pro-tumorigenesis and tumour recurrence (7, 8).

The findings from our study supported the previously reported immunosuppressive effects of tumour resection (5).Firstly, we identified that an immunosuppressive phenotype prevailed in the immediate postoperative setting. This was characterised by a reduction in the frequency of expanded circulating Th1-like cells post-operatively with a subsequent decrease in IFN-γ production. Donlon et al, recently showed that systemic Th1 immune responses were dampened in the post-operative setting illustrated by a decrease in the circulating levels of Th1 cytokines such as IFN-γ and IL-12p40 (5).

Furthermore, our results suggested a potential role for ICs in propagating these observed immunosuppressive effects, as we noted an increase in the expression of PD-L1 on the surface of expanded circulating Th1-like immune cells *ex vivo* one-week post-surgery. Previously, the immune checkpoint expression profile was evaluated on circulating T cells immediately post-surgery and identified that TIM-3 was upregulated on T cells immediately post-surgery before returning to baseline by week 6 (5). In contrast, PD-L1 immune checkpoint expression was not upregulated on circulating T cells immediately post-surgery and was indeed found to be downregulated on circulating T cells by week 6 post-surgery (5). However, this study did not profile immune checkpoint expression on T helper subsets, which may explain the discrepancies (5). In summary, these results together might indicate that the activation of Th1 immunity post-surgery might be inhibited by upregulation of PD-L1 immune checkpoint suggesting that blockade of the PD-1-PD-L1 axis may enhance Th1 immune responses post-surgery for this patient cohort. Moreover, the results from this study revealed that the cytotoxic ability of PBMCs was abrogated in the immediate post-operative setting by day 7. The reduced killing ability of PBMCs against radiosensitive and radioresistant tumour cells was observed, strengthening the hypothesis that tumour resection dampens Th1 immunity and anti-cancer cytotoxic responses. This provided us with further rationale to test our hypothesis that targeting the PD-1/PD-L1 pathway might be useful in abrogating the observed surgery-induced immunosuppression.

Considering the systemic tumour-promoting environment in the body created during the post-operative period previously characterised by an increase in pro-survival tumour factors, pro-angiogenic and pro-inflammatory mediators, the ability to prevent or abrogate the suppression of anti-tumour immunity would be a useful tool in promoting cancer immune surveillance, preventing the growth of micrometastatic disease and reduce the risk of cancer recurrence (5, 7–9).

Th1 cells play a pivotal role in performing cancer immune surveillance and mediating anti-tumour immunity to firstly prevent tumour formation and secondly eradicate tumours (10). Key Th1 mediators include IFN-γ and GrB which are essential components in the direct killing of tumour cells and inducing tumour cell apoptosis (11). Furthermore, our findings identified a concomitant increase in circulating levels of IL-10 in cancer patients post-operatively, which continued to increase 6 weeks post-surgery. Prior studies have also identified an increase in immunosuppressive cytokines post-operatively in other cancer types (5). Treg cells are a key producer of this immunosuppressive cytokine which function to inhibit Th1 immune responses (12). Activation of the PD-1/PD-L1 pathway in Th1 cells mediates their conversion into Treg cells *via* reduced STAT activation (13). Our findings, identifying an increase in PD-L1 expression on Th1 cells in parallel with an increase in the circulating levels of IL-10 in the immediate postoperative may suggest that the PD-1 axis is a mechanism for decreasing Th1 cells and increasing Treg cells post-operatively. Shigematsu et al (14), demonstrated that Treg cells suppressed cytotoxic lymphocyte mediated-killing of tumour cells. The authors observed that co-culturing Treg cells with cytotoxic lymphocytes and tumor cells decreased tumor cell killing and depletion of Treg cells in the co-culture system enhanced cytotoxic lymphocyte mediated-tumor cell killing (14).

In our study, we also found that several immune checkpoints including PD-1, PD-L1 and CTLA-4 were expressed at significantly higher levels on the surface of 5-day expanded post-operative Th1 and Th1/17 cells compared with 5-day expanded pre-operative T cells. This might suggest that post-operative T cells may be more prone to T cell exhaustion, creating a rationale for administering ICB in the post-operative setting as the target of ICB would be more highly expressed. Additionally, this potentially implicates the PD-1/PD-L1 pathway as a mediator of the decrease in anti-tumour Th1 cells and reduced cytotoxic ability of expanded circulating lymphocytes. Complementary findings were identified in a lung cancer study, where PD-1 expression was upregulated on T cells following surgical resection (9).

Th1/17 immune cells have potent anti-tumour properties which combine the cancer-fighting properties of Th1 cells and the ability of Th17 cells to self-renew and regenerate (15). In our study, single agent ipilimumab significantly decreased PD-1 expression on the surface of Th1/17 cells. The reduction in PD-1 expression may prevent inhibition or exhaustion of these potent anti-tumour cells.

The landmark CheckMate 577 phase III trial found that adjuvant nivolumab significantly improved patient outcomes, doubling disease-free survival following surgical resection of oesophageal or gastroesophageal cancers compared to placebo in patients who had received neoadjuvant chemoradiotherapy (6). Additionally, the adverse effect profile was favourable (6). Our findings complement those of that trial, providing a mechanistic rationale for the beneficial effects reported in CheckMate 577, namely by identifying postoperative immunosuppression and reduced anti-tumour cytotoxic ability of lymphocytes, which is abrogated by the addition of ICB. We found that nivolumab had significantly attenuated the depletion of Th1 cells and enhanced the cytotoxic ability of expanded circulating PBMCs in the immediate postoperative setting. A reduction in the cytotoxic ability of lymphocytes in the immediate post-operative setting may likely facilitate immune evasion of tumour cells, formation of micrometastatic deposits and tumour recurrence. Our study shows that use of ICB can enhance lymphocyte-mediated killing of tumour cells both radiosensitive and radioresistant tumour cells, which is critical given the high level of radiation resistance in oesophageal (16) and gastric cancer patients (17).

Of note we also observed that anti-PD-1 therapy enhanced anti-tumour Th1-like immunity prior to surgery. The beneficial effect of anti-PD-1 therapy in the neoadjuvant setting complements and supports the favourable findings from clinical trials that led to the FDA approval of nivolumab in combination with chemotherapy in the neoadjuvant setting in oesophagogastric junctional carcinomas providing supporting data for its efficacy. Our goal was to ask the question if ICB might also be advantageous in the adjuvant setting for promoting anti-tumour Th1-like immunity and abrogate the immunosuppressive effects surgery has on anti-tumour Th1 responses which we found it to be the case.

An interesting caveat we observed in this study was that ICB only increased IFN-γ and granzyme B production in expanded PBMCs in only a subset of patients. This finding is reminiscent of clinical response rates in that only a subset of patients will respond to ICB. Correlative analysis between ICB-mediated IFN-γ and GrB production by expanded PBMCs with immune factors in patients’ serum uncovered that patient’s with a higher level of circulating IFN-γ on POD 0 correlated positively with increased production of IFN-γ and GrB by PBMCs on POD 0. This observation suggests that these patients may have been pre-poised to have a better response to ICB perhaps because they had a higher level of immune activation against the tumour indicated by increased IFN-γ levels in the blood.

Acknowledging that the numbers are too low to draw any conclusions, we noted an interesting observation in the three patient-derived PBMC samples that did not respond to nivolumab, characterised by the absence of a nivolumab-mediated increase in IFN-γ production by PBMCs *ex vivo*. Responders were characterised by an increase in IFN-γ production by PBMCs *ex vivo*. Non-responders had an overall higher expression of CTLA-4 on CD4^+^Th1-like cells compared with responders to nivolumab on POD 0 (14.0 vs 4.0%) (data not shown). Additionally, in all three patients that did not respond to nivolumab the expression of CTLA-4 on CD4^+^ Th1 cells was increased after nivolumab treatment on POD 0 (14.0 and 23.0%, n=3), an effect that was not observed in patients who responded to nivolumab (4.0 vs. 3.0%, n=7) (data not shown). This may suggest that nivolumab-mediated upregulation of CTLA-4 may be one mechanism inhibiting the nivolumab-mediated increase in IFN-γ production by PBMCs *ex vivo*. This supports a rationale for dual blockade with nivolumab and ipilimumab to potentially help overcome these inhibitory effects on IFN-γ production by PBMCs. In a previous study published by our lab, we reported that dual nivolumab-ipilimumab treatment increased production of IFN-γ more substantially than single agent alone in oesophageal adenocarcinoma patient-derived T cells *ex vivo (* 18).

Our findings support existing literature in the creation of a tumour-promoting, immunosuppressive environment within the body following major OGJ oncologic surgery, including a depletion of circulating Th1 cells, reduced anti-cancer cytotoxic ability of lymphocytes and upregulation of immune checkpoint molecules (19). Additionally, we found that the use of ICB within the post-operative setting attenuated the surgery-mediated reduction in Th1 cytotoxic immunity. Given the poor prognosis associated with OGJ cancers, and the recent beneficial effects identified using ICB, the immediate post-operative period represents a viable therapeutic window of opportunity. This study underlines the immunosuppressive window created by surgery in the post-operative setting and the potential for ICB to harness the surgery-induced wound healing response that tends to be tumour-promoting and skew it towards a Th1-mediated immune response to prevent cancer recurrence and improve survival outcomes.

## Data availability statement

The original contributions presented in the study are included in the article/**Supplementary Materials**. Further inquiries can be directed to the corresponding author.

## Ethics statement

The studies involving human participants were reviewed and approved by Tallaght University Hospital/St James Hospital. The patients/participants provided their written informed consent to participate in this study.

## Author contributions

MD and CG contributed equally to this work and share first authorship. MD, CG, and ND wrote the manuscript. MD and ND conceptualized the study. MD, CG, FO’C, BM, EM, AS, ND, JP, JL, SGM, NLL, MGD and JY-T carried out experiments and data analysis. FO’C, EM, SR, ER, MGD, NLL, SGM, CB, LQ, CH, ET, EP, WB, JL, NR, CD, JR contributed to sample acquisition. All authors contributed to the article and approved the submitted version.

## References

[1] Donlon NE Power R Hayes C Reynolds J. v. Lysaght J. Radiotherapy, immunotherapy, and the tumour microenvironment: turning an immunosuppressive milieu into a therapeutic opportunity. Cancer Lett (2021) 502:84–96. doi: 10.1016/j.canlet.2020.12.045 33450360

[2] Bakos O Lawson C Rouleau S Tai LH. Combining surgery and immunotherapy: turning an immunosuppressive effect into a therapeutic opportunity. J Immunother Cancer (2018) 6. doi: 10.1186/s40425-018-0398-7 PMC6122574 30176921

[3] Yanagihara A Kagamu H Sakaguchi H Ishida H Nitanda H. Immunological impact of surgery in NSCLC patients. Ann Oncol (2019) 30:v587. doi: 10.1093/annonc/mdz258.006

[4] Tang F Tie Y Tu C Wei X. Surgical trauma-induced immunosuppression in cancer: recent advances and the potential therapies. Clin Transl Med (2020) 10:199–223. doi: 10.1002/ctm2.24 32508035 PMC7240866

[5] Donlon NE Davern M Sheppard AD O'Connell F Dunne MR Hayes C. The impact of esophageal oncological surgery on perioperative immune function; implications for adjuvant immune checkpoint inhibition. Front Immunol (2022) 13:823225. doi: 10.3389/fimmu.2022.823225 35154142 PMC8829578

[6] Tohme S Simmons RL Tsung A. Surgery for cancer: a trigger for metastases. Cancer Res (2017) 77:1548–52. doi: 10.1158/0008-5472.CAN-16-1536 PMC5380551 28330928

[7] Donlon NE Davern M Hayes C Power R Sheppard AD. The immune response to major gastrointestinal cancer surgery and potential implications for adjuvant immunotherapy. Crit Rev Oncol Hematol (2022) 175. doi: 10.1016/j.critrevonc.2022.103729 35662586

[8] Xu HM. Th1 cytokine-based immunotherapy for cancer. Hepatobiliary Pancreat Dis Int (2014) 13:482–94. doi: 10.1016/S1499-3872(14)60305-2 25308358

[9] Velotti F Barchetta I Cimini FA Cavallo MG. Granzyme b in inflammatory diseases: apoptosis, inflammation, extracellular matrix remodeling, epithelial-to-Mesenchymal transition and fibrosis. Front Immunol (2020) 11:2828. doi: 10.3389/fimmu.2020.587581 PMC7686573 33262766

[10] Sakaguchi S Wing K Onishi Y Prieto-Martin P Yamaguchi T. Regulatory T cells: how do they suppress immune responses? Int Immunol (2009) 21:1105–11. doi: 10.1093/intimm/dxp095 19737784

[11] Cai J Wang D Zhang G Guo X. The role of PD-1/PD-L1 axis in treg development and function: implications for cancer immunotherapy. Onco Targets Ther (2019) 12:8437–45. doi: 10.2147/OTT.S221340 PMC6800566 31686860

[12] Shigematsu Y Hanagiri T Shiota H Kuroda K Baba T. Immunosuppressive effect of regulatory T lymphocytes in lung cancer, with special reference to their effects on the induction of autologous tumor-specific cytotoxic T lymphocytes. Oncol Lett (2012) 4:625. doi: 10.3892/ol.2012.815 23205074 PMC3506612

[13] Sperk M van Domselaar R Neogi U. Immune checkpoints as the immune system regulators and potential biomarkers in HIV-1 infection. Int J Mol Sci (2018) 19. doi: 10.3390/ijms19072000 PMC6073446 29987244

[14] Chatterjee S Daenthanasanmak A Chakraborty P Wyatt MW Dhar P. CD38-NAD+Axis regulates immunotherapeutic anti-tumor T cell response. Cell Metab (2018) 27:85–100.e8. doi: 10.1016/j.cmet.2017.10.006 29129787 PMC5837048

[15] Kelly RJ Ajani JA Kuzdzal J Zander T Van Cutsem E. Adjuvant nivolumab in resected esophageal or gastroesophageal junction cancer. New Engl J Med (2021) 384:1191–203. doi: 10.1056/NEJMoa2032125 33789008

[16] Kato F Monma S Koyanagi K Kanamori J Daiko H. Long-term outcome after resection for recurrent oesophageal cancer. J Thorac Dis (2018) 10:2691. doi: 10.21037/jtd.2018.05.17 29997931 PMC6006052

[17] Yoo CH Noh SH Shin DW Choi SH Min JS. Recurrence following curative resection for gastric carcinoma. Br J Surg (2000) 87:236–42. doi: 10.1046/j.1365-2168.2000.01360.x 10671934

[18] Mikuni H Yamamoto S Kato K. Nivolumab for the treatment of esophageal cancer. Expert Opin Biol Ther (2021) 21:697–703. doi: 10.1080/14712598.2021.1904887 33736560

[19] Davern M Donlon NE O'Connell F Gaughan C O'Donovan C. Acidosis significantly alters immune checkpoint expression profiles of T cells from oesophageal adenocarcinoma patients. Cancer Immunol Immunother (2023) 72. doi: 10.1007/s00262-022-03228-y PMC9813044 35708739

