# Supplementary material for: PD-1 blockade attenuates surgery-mediated immunosuppression and boosts Th1 immunity perioperatively in oesophagogastric junctional adenocarcinoma
Source: Front Immunol. 2023 Jun 9;14:1150754. doi: 10.3389/fimmu.2023.1150754 (PMC10288841; doi:10.3389/fimmu.2023.1150754)
Supplement: Supplementary file 1 [file Image_1.pdf]

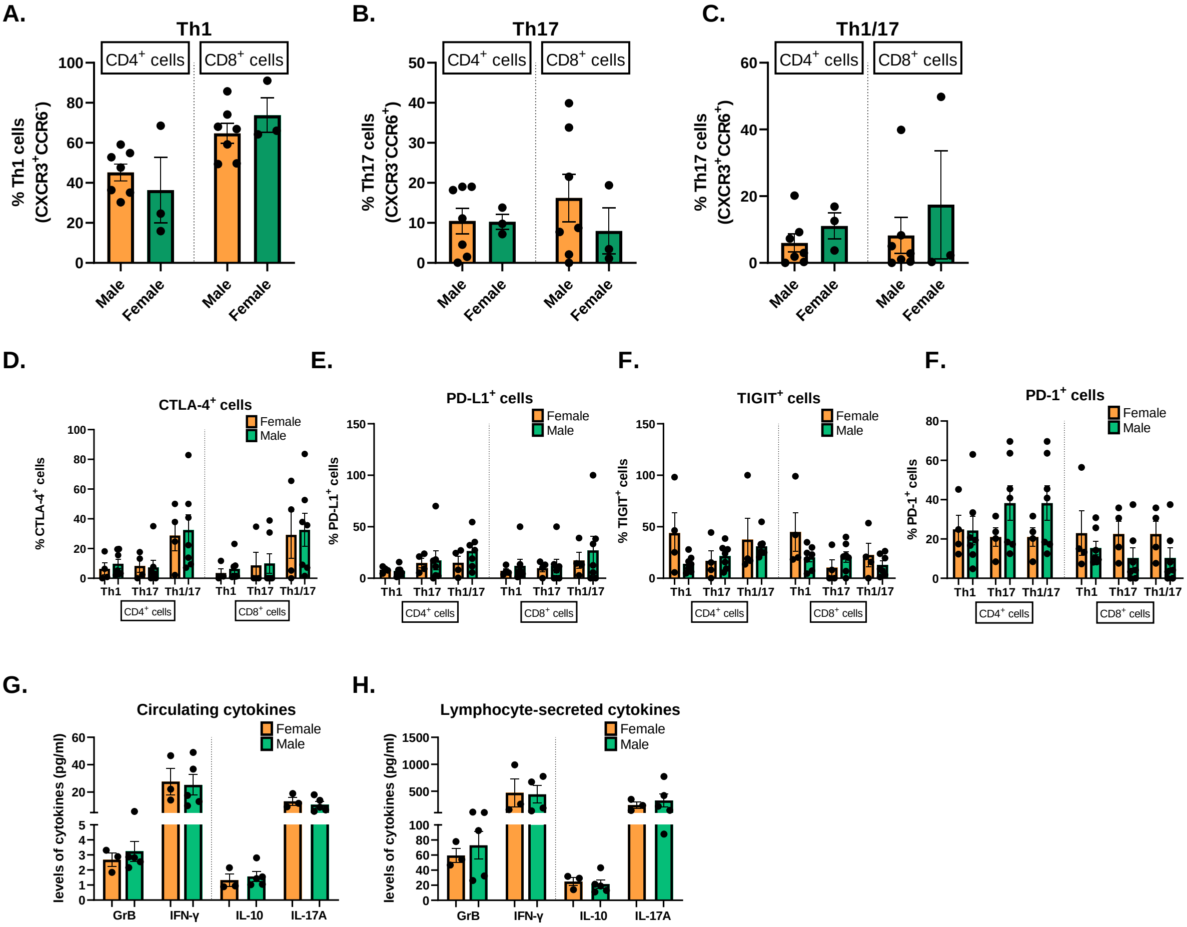

Figure S1: Immunological differences between female and male UGI cancer patients. The levels of circulating Th1- (A.), Th17- (B.) and Th1/17-like (C.) T cells was assessed in female versus male UGI cancer patients was determined by flow cytometry. The expression of immune checkpoints CTLA-4 (D.), PD-L1 (E.), TIGIT (F.) and PD-1 (G.) was measured on the surface of circulating Th1-,Th17- and Th1/17-like T cells in female and male UGI cancer patients by flow cytometry. (H.) and (I.) showcases the levels of circulating cytokines and the levels of 5-day expanded lymphocyte-secreted cytokines using multi-plex ELISA in female versus male patients. Cohort size: n=5 female and n=7 male UGI cancer patients. One-way ANOVA statistical analysis was conducted, $p<0.05$.
